# Expression of tenocyte lineage-related factors in regenerated tissue at sites of tendon defect

**DOI:** 10.1007/s00776-014-0684-2

**Published:** 2014-12-27

**Authors:** Takaaki Omachi, Tadahiro Sakai, Hideki Hiraiwa, Takashi Hamada, Yohei Ono, Motoshige Nakashima, Shinya Ishizuka, Tetsuya Matsukawa, Tomoyuki Oda, Akira Takamatsu, Satoshi Yamashita, Naoki Ishiguro

**Affiliations:** Department of Orthopaedic Surgery, Nagoya University Graduate School of Medicine, 65 Tsurumai-Cho, Showa-Ku, Nagoya, 466-8550 Japan

## Abstract

**Background:**

The healing mechanism of ruptured or injured tendons is poorly understood. To date, some lineage-specific factors, such as scleraxis and tenomodulin, have been reported as markers of tenocyte differentiation. Because few studies have focused on tenocyte lineage-related factors with respect to the repaired tissue of healing tendons, the aim of this study was to investigate their expression during the tendon healing process.

**Methods:**

Defects were created in the patellar tendons of rats, and the patellae and patellar tendons were harvested at 3 days and at 1, 2, 3, 6, 12, and 20 weeks after surgery. They were studied using micro-computed tomography, and paraffin-embedded sections were then prepared for histological evaluation. Reverse transcription-polymerase chain reactions were performed to analyze the expression of genes related to the tenocyte lineage, chondrogenesis, and ossification.

**Results:**

Repaired tissue became increasingly fibrous over time and contained a greater number of vessels than normal tendons, even in the later period. Safranin O staining revealed the existence of proteoglycan at 1 week and its persistence through 20 weeks. Ossification was detected in all tendons at 12 weeks. The expression of tenocyte lineage-related genes was high at 1 and 2 weeks. Chondrogenic genes were up-regulated until 6 weeks. Runt-related transcription factor 2, an osteogenic gene, was up-regulated at 20 weeks.

**Conclusions:**

In our tendon defect model, cells participating in the tendon healing process appeared to differentiate toward tenocyte lineage only in the early phase, and chondrogenesis seemed to occur from the early phase onward. To improve tendon repair, it will be necessary to promote and maintain tenogenesis and to inhibit chondrogenesis, especially in the early phase, in order to avoid erroneous differentiation of stem cells.

## Introduction

The healing mechanism of ruptured or injured tendons is poorly understood. Even over periods of long-term observation, the structure and strength of repaired tissue does not show full recovery and a return to normal. Many authors have studied tendon healing [1, 2]. Sharma et al. [3] reported that tendon healing occurred in three overlapping stages: the inflammatory stage (from injury to a few days after injury), the remodeling stage (a few days to 6 weeks after injury), and the modeling stage (6 weeks to over 1 year after injury). One of the early events of wound healing is angiogenesis, in which neovascularization prompts the delivery of inflammatory cells and fibroblasts to the wound site. In wounds, hypoxia-inducible factor-1 (HIF1) plays an important role in the intracellular hypoxic response. HIF1 is a heterodimeric transcription factor composed of an alpha and a beta subunit. In cultured fibroblasts undergoing cyclical strain, overuse has been shown to strongly induce the alpha subunit of HIF1 (HIF1A) [4]. HIF1 is an important up-regulator of vascular endothelial growth factor (VEGF), which promotes the formation of new blood vessels after tendon injury [5–9].

In a tendon window injury model, Lui et al. [10, 11] reported that fibrous tendinous tissue as well as chondrocyte phenotype and ectopic ossification were observed in collagenase-damaged tendons, and speculated that erroneous differentiation of stem cells may have occurred. Some stem cells have been demonstrated to form tendinous tissue in vitro and in vivo. These cells, which have been detected in the synovium, bone marrow, adipose, and tendon, are reported to be multipotent [12]. They are believed to play a role in tendon healing, although it remains unknown whether they participate in healing responses for tendon injuries in vivo. To date, some lineage-specific factors, including scleraxis (SCX) and tenomodulin (TNMD), have been reported as markers of tenocyte lineage differentiation. SCX is a member of the basic helix-loop-helix family of transcription factors [13], and is expressed in tendon progenitors in the embryonic phase. It also positively regulates the expression of TNMD in the tenocyte lineage [14]. TNMD is a type II transmembrane protein that is specifically expressed in dense connective tissue, including tendons and ligaments [15, 16]. Mice lacking TNMD display severely reduced tenocyte proliferation in tendons at birth and a disrupted collagen fibril structure in adulthood [17]. Some chondrogenic factors have been detected in Achilles [18] and rotator cuff tendinopathy [19]. Research has revealed that SRY (sex-determining region Y)-box 9 (SOX9) is essential for chondrocyte differentiation and cartilage formation [20]. Type II collagen (Col2) and aggrecan (AGG) are two major components of the cartilage matrix, and their expression is regulated by SOX9 [21, 22]. In addition to chondrometaplasia, ectopic ossification can be detected in some damaged tendons [10, 11]. Type X collagen (Col10) is a marker of hypertrophic chondrocytes and an indicator of endochondral ossification [23]. In addition, runt-related transcription factor 2 (RUNX2) is a key lineage-specific regulator of progenitor cell growth and differentiation, which stimulates osteoblast maturation during bone formation [24].

The ability to improve the properties of repaired tissue is needed in order to promote better outcomes in the treatment of tendon disorders, as this tissue is produced and interposed at the injury site irrespective of whether the treatment for tendon ruptures is surgical or conservative. Few studies have focused on factors related to tenocyte lineage in repaired tissue of healing tendons. The aim of this study, therefore, was to analyze the expression of lineage-specific genes related to tenocytes, chondrogenesis, and ossification in repaired tissue at tendon defect sites using a rat model. Our hypotheses are as follows. First, even if angiogenesis occurs in the early phase of tissue repair at sites of surgically created tendon defects, the number of vessels would decrease to levels observed in normal tendons in the later period. Second, fibrous tendinous tissue with some chondrogenic change would arise, and remodeling toward normal tendinous tissue would occur in the later period. In addition, factors related to the tenocyte lineage would be kept up-regulated for a longer period to reflect the remodeling of repaired tissue.

## Methods

### Tendon defect model

**Fig. 1:**
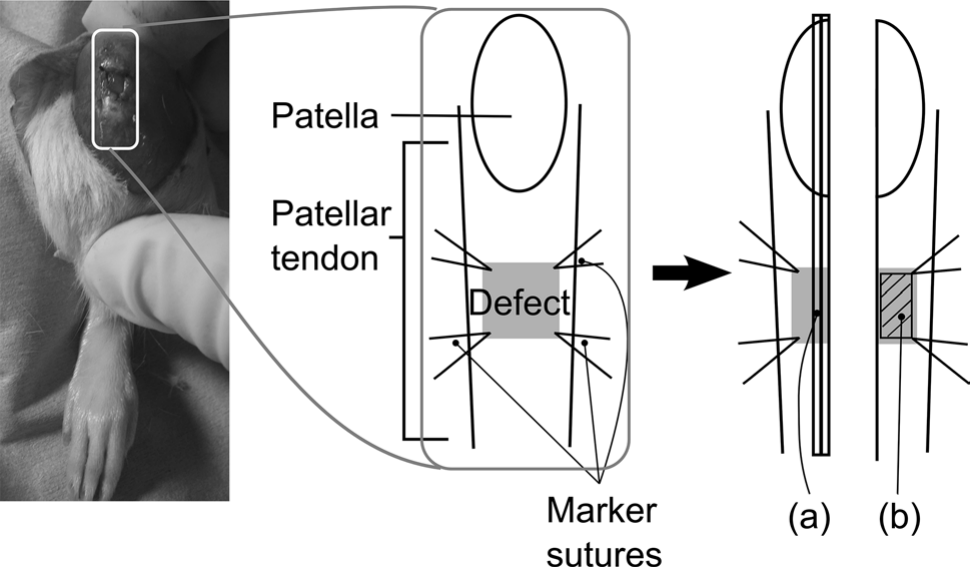
Tendon defect model. After harvesting, **a** half of the specimens were used to prepare paraffin-embedded sections, and **b** total ribonucleic acid (RNA) was extracted from repaired tissue of the remaining specimens

Forty-two male Sprague Dawley rats (12–15 weeks old, 380–470 g) were purchased from Charles River Laboratories Japan, Inc. The rats were anesthetized by intraperitoneal administration of 50 mg/kg pentobarbital (Nembutal, Sumitomo Dainippon Pharma, Japan). Skin incisions were made longitudinally over the patellar tendons, and the synovium over the patellar tendons was removed. On the treated side, 2-mm^2^ defects were created in the patellar tendons. Marker sutures (6-0 nylon sutures) were inserted into the four corners of the defects (Fig. 1). On the contralateral side of the knees, the patellar tendons were explored only as sham operations. Skin incisions were closed with running 6-0 nylon sutures. The patellae and patellar tendons were harvested at 3 days and at 1, 2, 3, 6, 12, and 20 weeks after surgery (*n* = 6, each) and then immersed in RNAlater (Qiagen, Hilden, Germany) solution in microtubes. They were studied using micro-computed tomography (micro-CT). Following this, they were longitudinally divided down the center. Half of the tissue specimens were used to prepare paraffin-embedded sections (Fig. 1a), and the remaining were used to extract total ribonucleic acid (RNA) for performing reverse transcription-polymerase chain reactions (RT-PCR) (Fig. 1b). The experimental design was approved by our institutional animal study committee.

### General histology

Half of the patellae and patellar tendons were fixed with 10 % buffered formalin, and were then decalcified with ethylenediaminetetraacetic acid (EDTA) and embedded in paraffin. Next, 5-µm sections were prepared in the direction of the longitudinal axis, and the sections were deparaffinized and stained using hematoxylin-eosin, Van Gieson’s, and safranin O staining protocols. A BZ-9000 microscope (Keyence Corporation, Osaka, Japan) was used to acquire digital micrographs. The number of vessels within a section area of 1 mm^2^ was counted.

### Real-time RT-PCR

The remaining half of the patellar tendon tissue samples were immersed in RNAlater solution (Qiagen, Hilden, Germany), and the repaired tissue was excised from the area within marker sutures. The average (standard deviation: ±SD) weight of the samples was 14.3 (±6.0) mg for the treated tendons and 13.6 (±3.9) mg for the sham group. The samples were snap-frozen using liquid nitrogen and ground into powder. Total RNA was extracted using the RNeasy Mini Kit (Qiagen), according to manufacturer instructions. A total of 50 µl of water was used for the final extraction from each RNeasy Mini spin column. RNA samples were then quantified with Eppendorf BioPhotometer plus (Eppendorf, Hamburg, Germany) and TrayCell with a 1-mm cap (Hellma, Müllheim, Germany). The average (±SD) concentrations of the RNA samples were 40.4 (±28.8) µg/ml from the treated tendons and 15.5 (±7.9) µg/ml from the sham group. RNA samples were reverse-transcribed to synthesize single-stranded complementary deoxyribonucleic acid (cDNA) samples using the High Capacity cDNA Reverse Transcription Kit (Applied Biosystems, Foster City, CA), according to manufacturer instructions. A total of 10 µl of RNA samples was used for a 20-µl reaction. The genes of interest were as follows: HIF1A, VEGF (angiogenesis-related); SCX, TNMD, and type I collagen (Col1) (tenocyte lineage related); SOX9, AGG, and Col2 (chondrogenesis related); and RUNX2 and Col10 (osteogenesis related). The sequences of primer pairs for messenger RNA (mRNA) of the target genes were selected from databases such as UniSTS (http://www.ncbi.nlm.nih.gov/unists) or were designed using the Primer3Web (http://primer3.sourceforge.net/) software and synthesized by Nihon Gene Research Laboratories, Japan. The primer pair of HIF1A was designed and synthesized by Sigma-Aldrich Japan (Table 1). A total of 1 µl of the cDNA samples and 10 pmol of primer pairs was mixed using the LightCycler 480 DNA SYBR Green I Master Mix (Roche Diagnostics, Basel, Switzerland) for 20 μl reactions, following the recommended protocol. Real-time quantitative PCR was performed using a LightCycler 480 II (Roche Diagnostics) following the recommended protocol for the SYBR Green I Master Mix, and the threshold cycle (Ct) of each gene was determined. In brief, PCR cycles consisted of denaturation at 95 °C for 10 s, annealing at 60 °C for 10 s, and extension at 72 °C for 10 s. If contamination was suspected in the melting curve analysis, the measurement was repeated. Given the limited amount of sample material, only single measurements were carried out. Some PCR products were diluted 10-fold, from 10^−5^ to 10^−8^µg/ml, and used as standards. PCR of these products was performed to obtain standard curves, and efficiency values were calculated from the slopes of the products using LightCycler 480 software (Roche Diagnostics) (Table 1). The relative expression ratio of the target gene was calculated using the Pfaffl method [25], and the ratio was expressed in comparison to a reference gene.

**Table 1:** Primer sequences for RT-PCR

		Sequence	Efficiency
HIF1A	Forward	GAAAGAGCCCGATGCCCTGA	1.88
	Reverse	TTGGTCTTCAGTTTCCGTGTCATC	
VEGF	Forward	TTCAGAGCGGAGAAAGCATT	1.82
	Reverse	GAGGAGGCTCCTTCCTGC	
SCX	Forward	TCATCCCGACCGAGCCAGCA	1.96
	Reverse	CCGCAGGCTTCACCCACCAG	
TNMD	Forward	CCTACTGCTACCAAGGAGGTCG	1.95
	Reverse	AGGATAGGCTACTTGGACGCAA	
Col1	Forward	CCGTGCTTCTCAGAACATCA	1.84
	Reverse	CTTGCCCCATTCATTTGTCT	
Sox9	Forward	AGACCAGTACCCGCATCT	1.96
	Reverse	CGCTCCGCCTCCTCCAC	
AGG	Forward	TTGGAGCCGGAGACGACAGA	1.81
	Reverse	AGAGGCAGAGGGACTTTCGGT	
Col2	Forward	TTCCTCCGTCTACTGTCCACTGA	1.97
	Reverse	CTACATCATTGGAGCCCTGGAT	
RUNX2	Forward	CCGCACGACAACCGCACCAT	1.89
	Reverse	CGCTCCGGCCCACAAATCTC	
Col10	Forward	TTGACAAAGATGCCAGAAA	1.79
	Reverse	TACAAGGTGCTTTACCACAGCC	
GAPDH	Forward	GCAACTCCCATTCTTCCACC	2.00
	Reverse	CACCCTGTTGCTGTAGCCATA	

*HIF1A* alpha subunit of hypoxia-inducible factor-1, *VEGF* vascular endothelial growth factor, *SCX* scleraxis, *TNMD* tenomodulin, *Col1* type I collagen, *SOX9* SRY (sex-determining region Y)-box 9, *AGG* aggrecan, *Col2* type II collagen, *RUNX2* runt-related transcription factor 2, *Col10* type X collagen, *GAPDH* glyceraldehyde-3-phosphate dehydrogenase

$${\text{ratio}}\; = \;\frac{{(E_{\text{target}} )^{{\Delta {\text{Ct}}_{\text{target}} ({\text{control}}\; - \;{\text{sample}})}} }}{{(E_{\text{ref}} )^{{\Delta {\text{Ct}}_{{{\text{ref}}\;}} ({\text{control}}\; - \;{\text{sample}})}} }}$$

*E*_target_ is the real-time PCR efficiency of a target gene transcript; *E*_ref_ is the real-time efficiency of a reference gene transcript. In this study, the glyceraldehyde-3-phosphate dehydrogenase (GAPDH) gene was used as a reference gene. ΔCt_target_ is the Ct deviation of the value of [control (the sample of the sham side) − sample (the sample of the treated side)] of the target gene transcript. ΔCt_ref_ is the Ct deviation of the value of (control − sample).

### Micro-CT

Samples of the patellae and patellar tendons were immersed in RNAlater solution in microtubes. Micro-CT of these samples was then performed using the R_mCT System (Rigaku Corporation, Japan). The X-ray tube voltage was set at 90 kV, and the current was set at 150 µA. A sample gantry diameter of 55 mm was selected for 10× magnification. The lower half of the patella and the treated area of the patellar tendon were included in the region of interest. The volume data were converted into multi-page TIFF files using the ATLAS Volume file tiff converter. The multi-page TIFF files were opened using ImageJ (National Institutes of Health Research Services Branch) software. Grayscale images in the multi-page TIFF files were converted to black-and-white binary images, with the threshold set at 22000/38049. Three-dimensional images were reconstructed using the3D Viewer ImageJ plugin (http://3dviewer.neurofly.de/). Volumes of ectopic ossification were calculated using the Sync Measure 3D plugin.

### Statistics

The number of vessels for each time point was presented as the average ± SD. The numbers of all time points were compared by one-way analysis of variance (ANOVA) using PASW Statistics software (version 18.0; IBM Corporation, Armonk, NY, USA). The Bonferroni test was used as a post hoc test. Gene expression was analyzed using RT-PCR. ΔCt of the target gene was calculated using the following formula: Ct of the target gene − Ct of the internal reference gene (GAPDH). At each time point, ΔCt of the treated side was compared with that of the sham side using 2 way-ANOVA with ANOVA4 (http://www.hju.ac.jp/~kiriki/anova4/about.html). The *p* value was set at 0.05. The relative expression ratio of the target gene was calculated using the Pfaffl method, and indicated as the average ± SD. Volumes of ectopic ossification calculated from micro-CT are presented as the average for each time point, with the range provided in parentheses.

## Results

### General histology

**Fig. 2:**
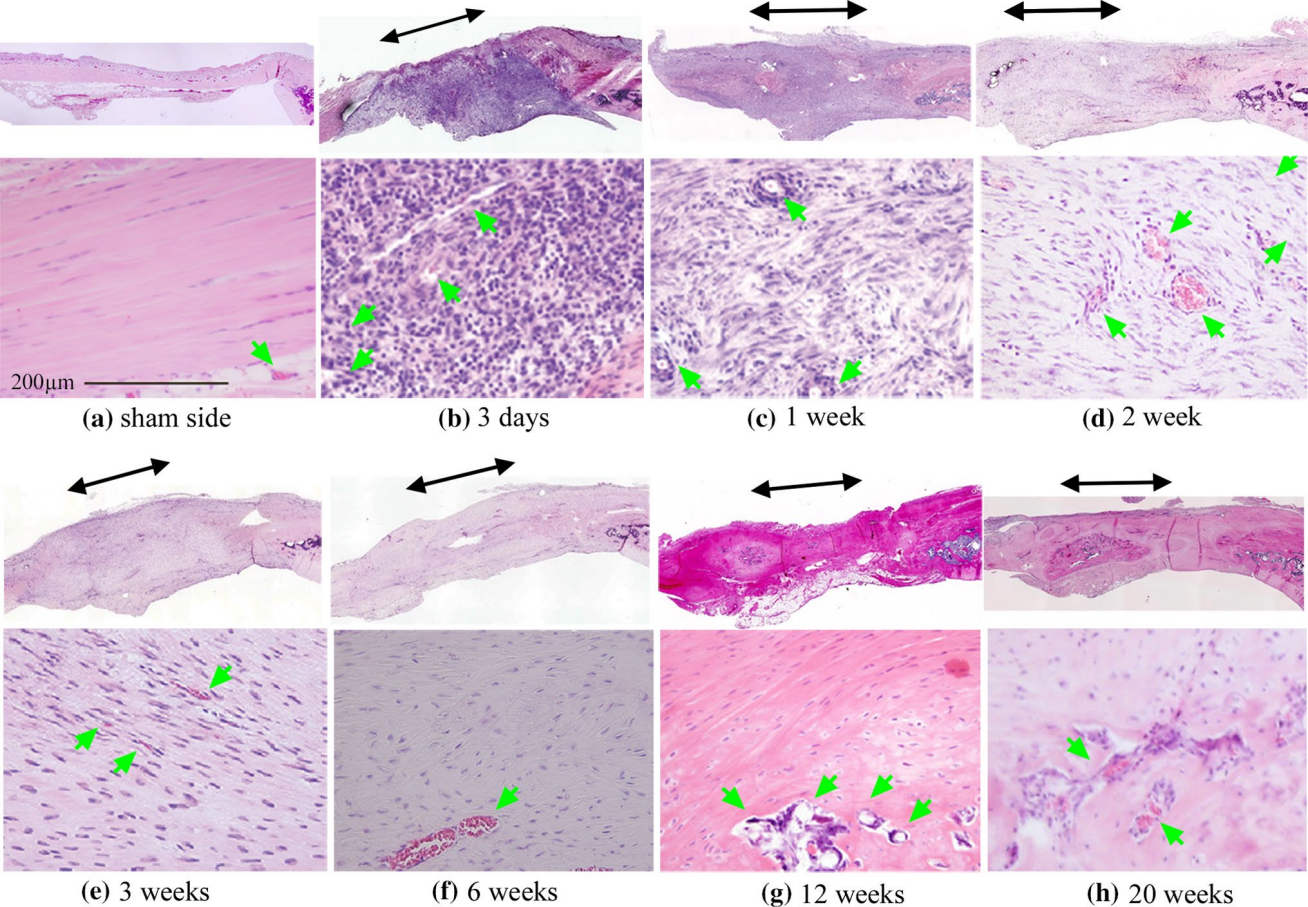
Hematoxylin-eosin-stained sections. The patellae are located on the *right* and the tendons (including repaired tissue) on the *left*. The location of the defects are indicated with *black double-ended arrows*. Vessels are indicated with *green arrowheads*. **a** On the sham side, vessels were present in the tissue around the tendon mid-substance, but not within it. **b** At 3 days, the defects were filled with repaired tissue, along with many cells and vessels. **c**–**g** While the tissue became more fibrous over time, its cellularity decreased. **g**–**h** At 12 and 20 weeks, ectopic ossification was present in all rats had

**Fig. 3:**
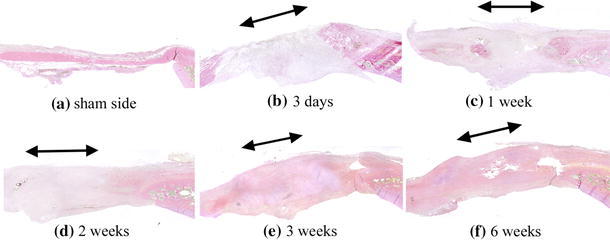
Sections with Van Gieson’s stain. **a** Collagen fibers were stained red on the sham side. **b**–**d** At 3 days and 1 and 2 weeks, the regenerated tissue at the tendon defect sites were not stained red. **e**–**f** They were partly stained red at 3 weeks and completely stained at 6 weeks

**Fig. 4:**
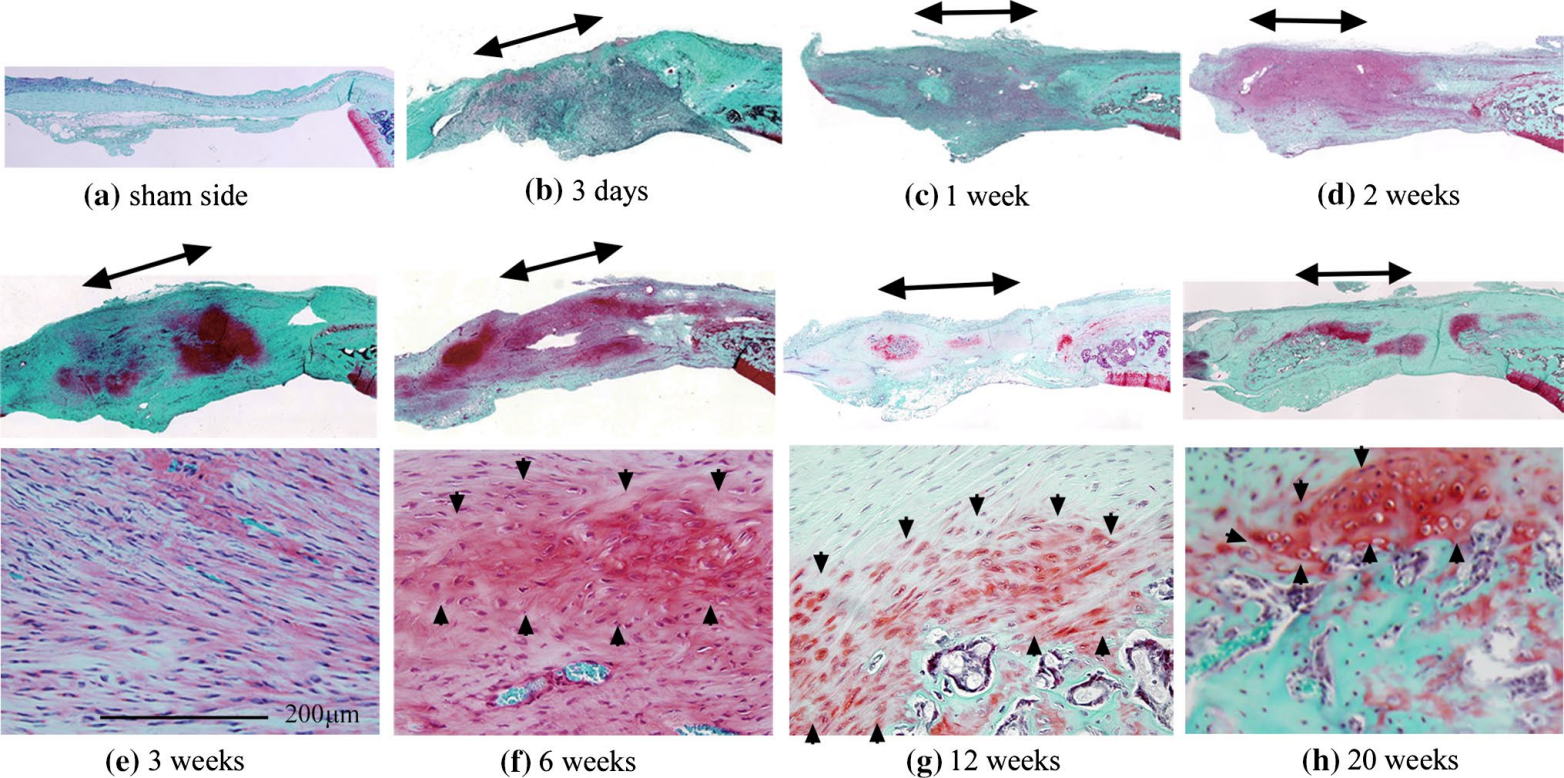
Safranin O-stained sections. **a** Tendons on the sham side were not stained with safranin O. **c** After 1 week, staining could be seen on sections of the treated tendons, including the injured site and surrounding tissue. **f** At 6 weeks, chondrocyte-like oval-shaped cells were detected, indicated by *black arrowheads*. **g**–**h** At 12 and 20 weeks, there appeared to be a transitional zone of bone, cartilage, and fibrous tissue around areas of ectopic ossification

On the sham side, the patellar tendons largely comprised dense connective tissue. The cells in the tendon resided between collagen fibers, and the number of cells was small (Fig. 2a). Vessels were present in the tissue around the tendon mid-substance, but not within it. After 3 days, the defects were filled with repaired tissue, along with a large number of cells and a few fibrous components (Fig. 2b). At 1 week, more fibrous components could be observed (Fig. 2c). While the tissue became more fibrous over time, its cellularity decreased (Fig. 2c–h). On the sections with Van Gieson’s staining, collagen fibers were stained red in the sham group (Fig. 3a). At 3 days and 1 and 2 weeks, the regenerated tissue at the tendon defect sites was not stained red (Fig. 3b–d), but was partially stained red at 3 weeks (Fig. 3e) and completely stained at 6 weeks (Fig. 3f). Tendons on the sham side were not stained with safranin O (Fig. 4a). After 1 week, sections of the treated tendons stained with safranin O (Fig. 4c) revealed the appearance of chondrometaplasia. Interestingly, the stained area included the treated site and the surrounding tissue (Fig. 4c–f). At 6 weeks, chondrocyte-like oval-shaped cells were detected in the safranin O-stained area (Fig. 4e). At 12 and 20 weeks, all rats exhibited ectopic ossification in the patellar tendons (Figs. 2g–h, 4g–h). The center of ossification appeared to be at the tendon defect site, although it extended beyond the treated site. There appeared to be a transitional zone of bone, cartilage, and fibrous tissue around the areas of ossification.

### Angiogenesis

**Fig. 5:**
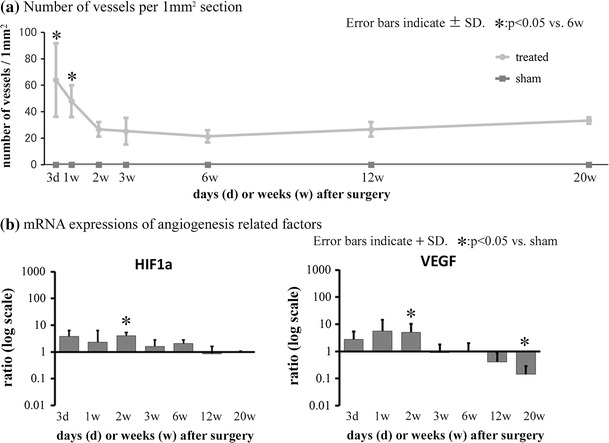
Angiogenesis-related factors. **a** The number of vessels within a 1-mm^2^ area of repaired tissue peaked at 3 days, with some vessels remaining even in the later period. There were no vessels in the tendon mid-substance on the sham side. **b** Reverse transcription-polymerase chain reaction (RT-PCR) detected messenger RNA (mRNA) upregulation of the alpha subunit of hypoxia-inducible factor-1 (HIF1A) and vascular endothelial growth factor (VEGF) at 2 weeks

The number of vessels within a 1-mm^2^ area of the repaired tissue was determined (Fig. 5a), and showed a peak at 3 days (64.0 ± 32.0 vessels). At 2 weeks, the number was reduced (26.7 ± 5.7 vessels), and it did not change remarkably thereafter. However, the number of vessels remained higher than that of the sham side.

### Angiogenesis-related factors

RT-PCR revealed that VEGF mRNA was up-regulated in the treated group compared to the sham group at 2 weeks (5.0 ± 5.3-fold, *p* < 0.05). HIF1A mRNA was also significantly up-regulated at 2 weeks (4.0 ± 1.3-fold, *p* < 0.05) (Fig. 5b).

### Tenocyte lineage-related factors

**Fig. 6:**
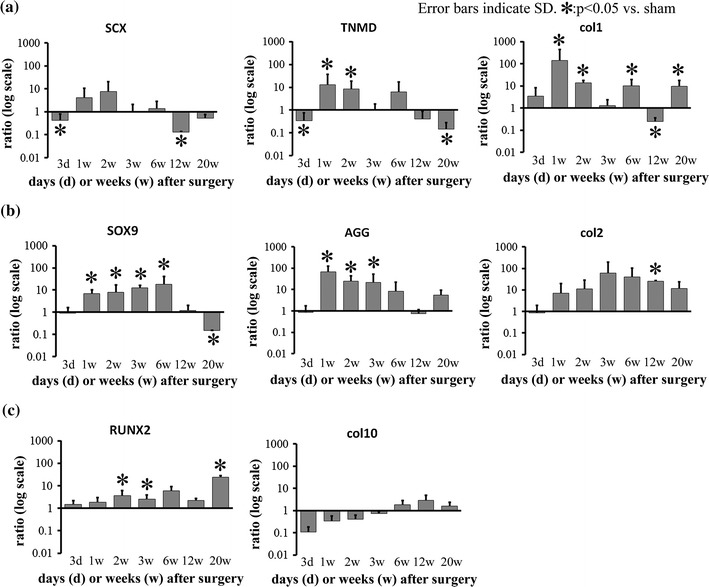
mRNA expression of lineage-specific factors. **a** Tenocyte lineage-related factors. mRNA expression of scleraxis (SCX) peaked at 2 weeks. Tenomodulin (TNMD) was up-regulated at 1 and 2 weeks. Type I collagen (Col1) was significantly up-regulated at 1 and 2 weeks. **b** Chondrogenesis-related factors. SRY (Sex-determining region Y)-box 9 (SOX9) was up-regulated at 1 week, and this upregulation persisted for 6 weeks. Aggrecan (AGG) was up-regulated at 1, 2, and 3 weeks. Significant mRNA upregulation of type II collagen (Col2) was detected at 12 weeks. **c** Osteogenesis-related factors. Runt-related transcription factor 2 (RUNX2) was up-regulated at 12 and 20 weeks

SCX expression in the treated group compared to the sham group peaked at 2 weeks (7.8 ± 12.9-fold, *p* = 0.16); however, its levels declined at 3 weeks and remained low until 20 weeks. TNMD mRNA expression was significantly up-regulated at 1 (13.1 ± 23.9-fold, *p* < 0.05) and 2 (8.3 ± 10.1-fold, *p* < 0.05) weeks. Col1 was up-regulated at 1 (139.6 ± 295.3-fold, *p* < 0.05), 2 (13.8 ± 3.7-fold, *p* < 0.05), 6 (9.9 ± 9.1-fold, *p* < 0.05), and 20 (9.5 ± 7.9-fold, *p* < 0.05) weeks (Fig. 6a).

### Chondrogenic factors

SOX9 mRNA expression was up-regulated in the treated group compared to the sham group at 1 week (7.1 ± 3.3-fold, *p* < 0.05), and remained up-regulated for 6 weeks (17.9 ± 22.3-fold, *p* < 0.05). AGG was up-regulated at 1 week (66.1 ± 56.7-fold, *p* < 0.05), and remained up-regulated for 3 weeks (22.0 ± 3.6-fold, *p* < 0.05). Significant upregulation of Col2 was detected at 12 weeks (25.8 ± 1.5-fold, *p* < 0.05) (Fig. 6b).

### Osteogenesis related factors

RUNX2 mRNA expression was up-regulated in the treated group compared to the sham group at 2 (3.7 ± 2.3-fold, *p* < 0.05), 3 (2.6 ± 1.4-fold, *p* < 0.05), and 20 (24.1 ± 4.7-fold, *p* < 0.05) weeks. No significant change was observed in Col10 mRNA expression (Fig. 6c).

### Ossification

**Fig. 7:**
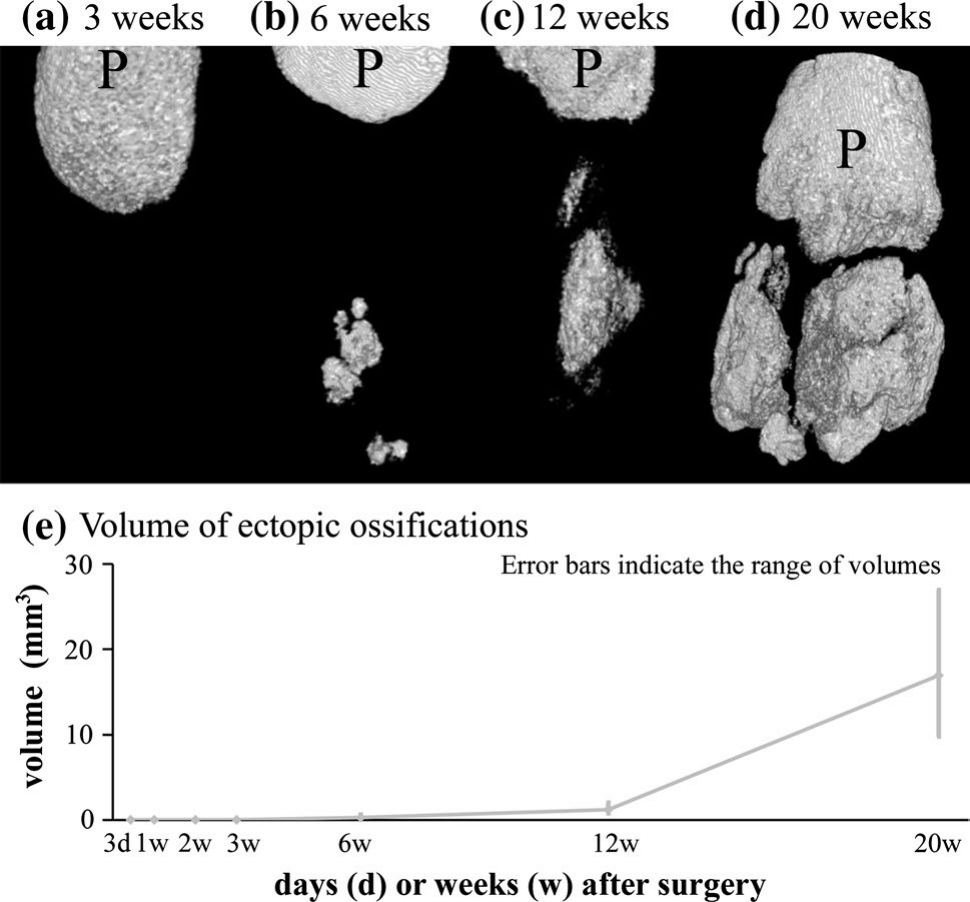
Micro-computed tomography (micro-CT) of the patellae and patellar tendons. Patellae are indicated as *P*. **a** No ectopic bone was detected at 3 weeks. **b** At 6 weeks, ectopic ossification was detected in 2 cases. **c**, **d** At 12 and 20 weeks, all tendons had ectopic ossification. **e** Mean volumes of ectopic ossification increased over time

Micro-CT revealed no ectopic bone formation on the sham side. On the treated side, ectopic bone was not detected at 3 weeks (Fig. 7a). At 6 weeks, ectopic ossification was observed in two cases (Fig. 7b). The volume of the ossification was 0.28 mm^3^ (range 0–0.60 mm^3^) (Fig. 7e). At 12 and 20 weeks, all tendons showed ectopic ossification (Fig. 7c, d). The volume of this ossification was 1.22 mm^3^ (range 0.74–2.01 mm^3^) at 12 weeks and 16.96 mm^3^ (range 9.76–26.95 mm^3^) at 20 weeks (Fig. 7e).

## Discussion

One of the early events of wound healing is neovascularization, which prompts the delivery of inflammatory cells and fibroblasts to the wound site. In this study, the numbers of vessels in repaired tissue were increased at 3 days and 1 week. The mRNA expression of HIF1A and VEGF in our RT-PCR assays was significantly up-regulated in the treated group compared to the sham group at 2 weeks, after which time no upregulation was detected. A normal tendon consists of dense connective tissue, with poorly formed blood vessels. It was expected that the number of vessels would fall to normal tendon levels in the later period. However, repaired tissue consisted of some vessels even in the later period. This could be one of the reasons for the incomplete recovery of repaired tissue. Vascularization into cartilage is a key mechanism for bone formation [26, 27], and sustained vascularity may be related to ectopic ossification.

Some types of mesenchymal stem cells are known to concentrate in tendon defect sites [28], and their differentiation patterns should determine the fate of the repaired tissue. However, the precise origins of these cells in the repaired tissue of damaged tendons have not been determined. If the tenocyte lineage-specific factors were up-regulated in the very early period, we could speculate that tenocytes or tendon stem cells were the main component of the stem cells for the formation of the regenerated tissue. But they were not up-regulated at 3 days, and thus the source of the stem cells was not clear from the results of this study. It was also expected that the gene expression of tenocyte lineage-related factors would remain up-regulated during tendon repair. In this study, the mRNA expression of SCX peaked at 2 weeks, and that of TNMD was significantly up-regulated at 1 and 2 weeks. Contrary to expectations, the gene expression of tenocyte lineage-related factors was up-regulated only in the earlier stages.

With regard to the chondrogenic factors, SOX9 mRNA expression was up-regulated in the treated group compared to the sham group at 1 week, and remained up-regulated for 6 weeks. AGG was up-regulated at 1 week and remained up-regulated for 3 weeks. The safranin O-stained paraffin-embedded sections revealed the presence of proteoglycan in repaired tissue at 1, 2, 3, and 6 weeks. As such, it seems that tissue undergoing repair is more inclined toward chondrogenic than tenogenic differentiation. One may speculate that this is one of the reasons for the failure of repaired tissue to achieve the characteristics of normal tendons even after 20 weeks.

Some growth factors have been reported to promote tendon differentiation. Lee et al. [28] noted that brief stimulation with bone morphogenetic protein (BMP) 12 in vitro was sufficient to induce the differentiation of bone marrow-derived mesenchymal stem cells into tenocytes, and that this phenotype was sustained in vivo. To date, however, no factor has been reported to up-regulate tendon lineage differentiation markers or maintain their upregulation for longer periods. The inhibition of chondrometaplasia may be important in preventing ectopic ossification, as ossification appears to emerge from areas of chondrometaplasia. Treatment in this case should be initiated in the early phase, as chondrometaplasia can be observed beginning in the first week of repair. Lui et al. [10, 11] demonstrated the presence of chondrometaplasia and ectopic ossification at the injury site in a rat collagenase-induced patellar tendon window injury model. The authors suggested that erroneous differentiation of stem cells may occur in the damaged tendon. In our study, RUNX2 mRNA expression was up-regulated at 2, 3, and 20 weeks, and micro-CT revealed ectopic ossification in the treated group after 6 weeks. There may be similarities among studies from the perspective of mechanical stress depreciation [18, 19] or factors such as BMPs [29].

This study did have some limitations. First, the number of samples in our study was small. Therefore, some RT-PCR data varied widely, and significance could not be demonstrated at some time points. Second, the volume of samples was also very small. We did not match the concentrations of mRNA in PCR study because some of them showed very low concentrations. Efficiencies of the primers used in this study were rechecked with dilution series of some of the cDNA samples, and they were almost identical to the range of the concentrations used in this study. We did not test the suitability of the GAPDH gene to act as a true internal standard for the sham-injured group, given the limited sample availability. Murphy and Polak reported that both HPRT and β-tubulin mRNA levels varied markedly between spontaneously differentiating and growth factor-supplemented murine embryonic stem cell cultures, while GAPDH expression remained relatively constant [30]. Although we did not utilize an in vitro study model with embryonic stem cells, the situations would appear to be similar, as stem cells and growth factors may be involved in the formation of repaired tissue, and thus it may be reasonable to consider GAPDH as an internal standard. Moreover, proteins were not analyzed due to the insufficient quantity (ten) and volume (just several milligrams) of samples. Analysis of proteins may become possible using a technique such as mass spectrometry capable of analyzing small samples.

In conclusion, some vessels were present in the repaired tissue at tendon defect sites even during the later period. Tenogenesis appeared to occur only in the early phase, while ectopic ossification emerged following chondrometaplasia that was maintained from the early phase to the late phase. To improve tendon healing, it will be necessary to promote tenogenesis and inhibit chondrogenesis (especially in the early phase) in order to avoid the erroneous differentiation of stem cells.
